# The Effect of Strength Training During Chemotherapy in Women With Breast Cancer on Serum Cytokine Concentrations and Skeletal Muscle Autophagy‐Related Proteins

**DOI:** 10.1002/ejsc.70213

**Published:** 2026-06-25

**Authors:** Emelie Strandberg, Olav Vikmoen, Karianne Vassbakk Svindland, Atle Wendel Scheie, Hege Nymo Ødemark, Anna Henriksson, Sveinung Berntsen, Truls Raastad

**Affiliations:** ^1^ Department of Public Health and Caring Sciences Uppsala University Uppsala Sweden; ^2^ Department of Physical Performance Norwegian School of Sport Science Oslo Norway; ^3^ Women's and Children's Health Uppsala University Uppsala Sweden; ^4^ Department of Sport Science and Physical Education University of Agder Kristiansand Norway; ^5^ Research Unit Sorlandet Hospital Kristiansand Norway

**Keywords:** anti‐cancer treatment, inflammation, interleukin 17, interleukin 6, muscle health

## Abstract

To evaluate whether strength training during chemotherapy alters (1) serum cytokines and (2) skeletal muscle autophagy‐ and heat‐shock‐related proteins in women with breast cancer. Exploratory analyses assessed associations between changes in physiological outcomes (muscle strength, cardiorespiratory fitness, and capillary density) and biomarker responses, and among cytokine changes. Women with breast cancer were randomized to strength training (*n* = 23) or usual care (*n* = 17). The training group completed supervised strength training twice weekly during chemotherapy; usual care maintained habitual physical activity. Assessments were performed pre‐chemotherapy (T0) and post‐chemotherapy/intervention (T1). Serum cytokines (IFN‐γ, IL‐1RA, IL‐6, IL‐8, IL‐10, IL‐17, MCP‐1, TNF‐α) were quantified, and muscle biopsies were analyzed for autophagy‐ and heat‐shock‐related proteins. In the strength training group, mean ± SD IL‐6 increased from 1.21 ± 0.47 to 1.69 ± 0.71 pg/mL and IL‐17 from 1.24 ± 0.25 to 2.18 ± 0.87 pg/mL. IFN‐γ also increased in the training group (14.4 ± 8.6 to 28.8 ± 17.3 pg/mL), however, the group×time interaction was not statistically significant. In the usual care group, IL‐6 and IL‐17 decreased. No changes were observed in other cytokines or in autophagy‐ or heat‐shock‐related proteins. Exploratory analyses showed no associations between changes in strength and autophagy proteins, cardiorespiratory fitness and cytokines, or capillary density and cytokines. Strength training during chemotherapy did not alter selected skeletal muscle autophagy‐ or heat‐shock‐related proteins, despite group differences in IL‐6 and IL‐17 over time. The clinical and mechanistic significance of these cytokine shifts remains uncertain and should be evaluated in larger studies with additional time points and complementary immune and muscle outcomes.

## Highlights

- Strength training during chemotherapy increased serum IL‐6 and IL‐17 compared with usual care in women with breast cancer.
- IFN‐γ increased descriptively in the strength‐training group, but the group × time interaction was not statistically significant.
- No changes were detected in skeletal muscle autophagy‐ or heat‐shock‐related proteins after the intervention.
- The clinical and mechanistic significance of the observed cytokine shifts remains uncertain and warrants further study.

## 1 Introduction

Breast cancer remains one of the most prevalent cancers affecting women worldwide, and chemotherapy remains a key component of treatment that substantially improves survival (Arnold et al. 2022; Giaquinto et al. 2022). However, chemotherapy often leads to adverse effects beyond tumor targeting, impacting muscle health such as reduced muscle strength, changes in body composition, and impaired muscle repair and remodeling (Freedman et al. 2004; Saquib et al. 2007; Van Den Berg et al. 2017; Vance et al. 2011).

Chemotherapy is known to induce systemic and tissue‐specific inflammation, in part mediated by cytokines (Behranvand et al. 2022), and a dysregulation of cytokines can contribute to tissue damage (Elenkov et al. 2005). Circulating cytokines measured in serum are commonly used to assess systemic inflammation but may not capture skeletal muscle‐specific inflammatory or stress signaling. Elevated pro‐inflammatory cytokines such as interleukin‐6 (IL‐6) and tumor necrosis factor‐alpha (TNF‐α) are commonly associated with muscle catabolism and fatigue, prominent issues in cancer patients undergoing chemotherapy (Webster et al. 2020). Conversely, anti‐inflammatory cytokines such as interleukin‐10 (IL‐10) may offer protective effects against muscle degradation, highlighting a complex, bidirectional role of cytokine signaling in muscle health during cancer treatment (Cole et al. 2018; Pedersen and Febbraio 2012). In addition to systemic inflammation, chemotherapy may disrupt intracellular quality‐control systems in skeletal muscle. Autophagy, a cellular degradation process responsible for the clearance of damaged proteins and organelles, plays a vital role in maintaining muscle homeostasis, particularly under physiological stress such as chemotherapy or exercise (Ryter et al. 2013). Chemotherapy may alter regulation of this process, contributing to muscle wasting through accumulation of dysfunctional proteins and increased cellular stress (Thorburn et al. 2014; Tilija Pun et al. 2020). Heat shock proteins (e.g., HSP60 and HSP70) function as molecular chaperones involved in protein folding and protection against oxidative and cytotoxic stress (Calderwood et al. 2009; Sun et al. 2022). Because chemotherapy exposes skeletal muscle to repeated metabolic and inflammatory stress, changes in autophagy‐ and heat‐shock related proteins may provide insight into whether muscle is experiencing cellular strain.

Exercise performed during chemotherapy may modulate the inflammatory balance, however, in previous studies inconsistent findings (likely reflecting heterogeneity in cancer type, treatment regimens, sampling timing, and exercise prescription) have been reported, with both increases and decreases observed in pro‐ and anti‐inflammatory markers (de Hoop et al. 2024). Most trials to date have implemented combined aerobic and strength training interventions, making it difficult to isolate the specific effects of strength training. This distinction is important because aerobic exercise and strength training differ in contraction pattern, metabolic demand, and the signaling pathways they preferentially activate. Strength training is particularly relevant in the present clinical setting because it directly targets muscle mass and strength, outcomes that are negatively impacted by chemotherapy. To the authors' knowledge, strength training has been investigated as a standalone intervention during chemotherapy in only one previous trial, which also reported systemic inflammatory outcomes (Christensen et al. 2014). That study enrolled men with testicular germ cell cancer, which limits generalizability to women with breast cancer. In addition to cytokine signaling, muscle adaptations during chemotherapy may be influenced by intracellular pathways that regulate protein homeostasis and cellular stress responses. In healthy skeletal muscle, exercise is known to influence both autophagy‐ and heat‐shock‐related pathways as part of the adaptive response to contractile stress (Noble et al. 2008; Vainshtein and Hood 2016). Strength training may be particularly relevant in this context, as repeated high‐load contractile activity can engage pathways involved in protein quality control, repair, and cellular protection (Henstridge et al. 2016; Ribeiro et al. 2025). To date, autophagy‐ and heat shock‐related signaling in skeletal muscle in response to strength training performed concurrently with chemotherapy has not been characterized in any study, underscoring the need for further research.

We have previously shown, in the same cohort, that strength training during chemotherapy improves muscle strength and preserves muscle fiber cross‐sectional area (CSA) (Vikmoen et al. 2024). Cardiorespiratory fitness (VO_2peak_) and hemoglobin declined similarly in the strength‐training and control groups. However, strength training preserved mitochondrial enzyme content, prevented loss of type II fiber capillary density, and blunted increases in physical fatigue (Vikmoen et al. 2025). Building on these findings, the present study evaluated systemic inflammatory signaling and intramuscular cellular stress and protein quality‐control responses during chemotherapy. Systemic inflammation was assessed using serum cytokine concentrations, while intramuscular responses were assessed using autophagy‐ and heat‐shock‐related protein abundance in skeletal muscle (ATG5, LC3B1, p62, BCL2, HSP60, and HSP70).

Accordingly, this study aimed to investigate the effects of strength training during chemotherapy on: (1) serum cytokine concentrations, and (2) skeletal muscle autophagy‐ and heat‐shock‐related protein abundance in women with breast cancer. As exploratory aims, we also examined associations between changes in physiological outcomes and biomarker responses, including relationships between changes in muscle strength and autophagy‐related markers, changes in VO_2peak_ and cytokine levels, changes in skeletal muscle capillary density and cytokine levels, and interrelationships among cytokine changes.

## 2 Methods

### 2.1 Study Design

**FIGURE 1:**
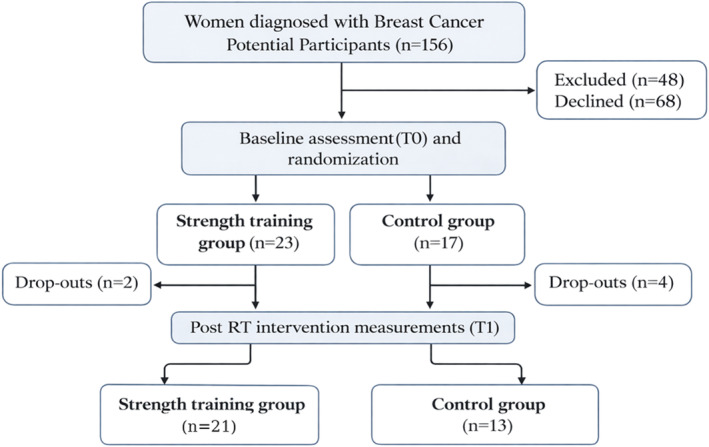
CONSORT flow diagram of participant recruitment, allocation, follow‐up, and inclusion in the present analyses. The diagram shows the number of patients screened for eligibility, excluded, randomized to strength training or usual care, followed during chemotherapy.

This study was designed as a two‐armed, randomized controlled trial including women with breast cancer undergoing chemotherapy. Participants were randomly assigned to one of two groups: A strength training (ST) group (*n* = 23) that engaged in supervised strength training during chemotherapy, or a usual care (CON) group (*n* = 17) that did not participate in structured exercise. Data were collected at two time points: before the initiation of chemotherapy (T0) and immediately after the completion of both chemotherapy and the training intervention (T1), approximately 16 weeks after start. Details on the recruitment process, randomization protocol, participant characteristics, and primary outcomes have previously been described (Strandberg et al. 2021; Vikmoen et al. 2024). A CONSORT flow diagram for this randomized controlled trial has been published previously (Vikmoen et al. 2024). For transparency and to improve readability of the present manuscript, a flow diagram is also included here, showing the number of patients screened, eligible, recruited, randomized, followed during chemotherapy, and included in the present analyses (Figure 1).

### 2.2 Ethics

The present study was approved by the Regional Ethical Review Board in Uppsala, Sweden (Dnr 2016/230/2) and registered in ClinicalTrials.gov (NCT04586517). The study was conducted in accordance with the Declaration of Helsinki. All participants provided written informed consent prior to participation, and personal data were handled in accordance with applicable GDPR regulations.

### 2.3 Participant Recruitment and Eligibility Criteria

Women newly diagnosed with stage I–III breast cancer were recruited from Uppsala University Hospital. To be eligible, participants had to be over 18 years of age, able to read and understand Swedish, and scheduled to receive (neo)adjuvant chemotherapy that included either taxanes, anthracyclines, or a combination of both. Individuals were excluded if they had significant limitations in performing basic activities of daily living, were affected by cognitive impairments or severe emotional instability, or had comorbidities likely to interfere with participation in physical training (e.g., advanced heart failure, chronic obstructive pulmonary disease, major orthopedic issues, or neurological conditions). Eligibility screening was conducted by the patient's surgical or oncology care team. A detailed description of the recruitment process and eligibility criteria has been published elsewhere (Strandberg et al. 2021; Vikmoen et al. 2024).

### 2.4 Randomization

After completion of baseline (T0) assessments and muscle biopsies, participants were randomized 1:1 to strength training or usual care. Randomization was stratified by treatment setting ((neo)adjuvant versus adjuvant), with separate allocation sequences for each stratum. An independent member of the research team (not involved in recruitment, training, or outcome assessments) prepared sequentially numbered, opaque, sealed envelopes containing group assignments (“strength training” or “usual care”). Envelopes were stored in a locked cabinet. After baseline testing was completed, the study coordinator opened the next envelope corresponding to the participant's treatment setting and informed the participant of group allocation. Due to the nature of the intervention, participants and the study coordinator were not blinded to group allocation; laboratory analyses were performed by personnel blinded to group assignment.

### 2.5 Sample Size

Power calculation has been described elsewhere (Strandberg et al. 2021; Vikmoen et al. 2024) and was based on expected changes in muscle fiber size. Muscle fiber size outcomes from the present trial have been reported previously (Vikmoen et al. 2024) and are not included here. Briefly, the original calculation indicated that 10 participants per group were required to achieve 80% power for the muscle fiber size endpoint. The study was designed with balanced randomization, with the intention of an even split between groups. Recruitment was stopped when the target sample size for the study had been reached. The cytokine and muscle protein outcomes reported in the present manuscript were secondary endpoints; given the number of outcomes assessed and missing data for some markers, the study may have been underpowered to detect modest between‐group differences over time.

### 2.6 Strength Training Program

The exercise protocol and muscle strength testing has been described elsewhere (Strandberg et al. 2021). Briefly, participants in the ST group performed strength training sessions twice per week, which included the exercises seated leg press, chest press, leg curl, seated row, leg extension, and seated overhead press with dumbbells. During the initial 2 weeks, participants underwent a familiarization phase using lighter loads. Thereafter, participants completed one weekly session using heavier loads corresponding to approximately 6RM and one weekly session using moderate loads corresponding to approximately 10RM. Rest intervals between sets were 2 min during the 6RM sessions and 1 min during the 10RM sessions. Training loads were adjusted regularly throughout the intervention based on repeated RM testing. In each exercise, the final set was performed to volitional failure, whereas the earlier sets were terminated when the target repetition range had been completed. Thus, volitional failure was used to ensure sufficient effort in the final set rather than as a requirement for all sets. In contrast, participants in the CON group were advised to maintain their usual physical activity levels and refrain from initiating any new strength training routines during chemotherapy. The overall adherence for the training period was assessed by averaging the adherence of all sessions. The average adherence to the training protocol was 72% ± 16% (range 41%–99%) (Vikmoen et al. 2025, 2024).

### 2.7 Cardiorespiratory Fitness

Cardiorespiratory fitness was assessed as VO_2peak_ using a graded treadmill exercise test based on a modified Balke protocol. The test started at a speed of 4 km/h with a 2% incline, which increased by 1% each minute until reaching a 12% incline. After that point, the speed increased by 0.5 km/h every minute until the participant reached volitional exhaustion. Oxygen consumption and minute ventilation were continuously measured using an oxygen analyzer (Vmax Encore system, Carefusion, CA).

### 2.8 Cytokine Analysis

Venous blood samples were collected at T0 and T1. Post‐intervention testing (T1) was scheduled ≥ 48 h after the final supervised strength‐training session to minimize acute exercise effects. Participants were additionally instructed to avoid any strenuous physical activity for 24 h prior to each testing visit to reduce potential confounding from unsupervised exercise. Participants were also instructed to avoid smoking and alcohol 24 h before sampling. Samples were processed immediately, and serum was stored at −80°C until further analysis. Serum cytokine levels of interleukin (IL)‐1 receptor antagonist (IL‐1RA), IL‐1β, IL‐6, IL‐8, IL‐10, IL‐17, interferon‐γ (IFN‐γ), monocyte chemoattractant protein‐1 (MCP‐1) and tumor necrosis factor alpha (TNF‐α) were quantified using a customized V‐PLEX assay (Mesoscale Discovery, Rockville, MD, USA). Analyses were performed on a SECTOR Imager 2400 instrument at Vitas Analytical Laboratory AS in Oslo, with technicians blinded to participant clinical information. IL‐1β values were below the lower limit of quantification (LLOQ; 0.29 pg/mL) for a substantial proportion of samples and were therefore not included in statistical analyses. Assay‐provided LLOQs were IFN‐γ: 1.65 pg/mL, IL‐6: 0.12 pg/mL, IL‐8: 0.14 pg/mL, IL‐10: 0.12 pg/mL, IL‐17: 0.70 pg/mL, and TNF‐α: 0.19 pg/mL; assay‐provided upper limits of quantification (ULOQ) were IL‐1RA: 4590 pg/mL and MCP‐1: 5440 pg/mL.

### 2.9 Muscle Biopsy Procedure

Muscle biopsy procedure has been described previously (Vikmoen et al. 2024). Briefly, muscle biopsies were obtained from the mid‐portion of the *m. vastus lateralis* under local anesthesia (Xylocain adrenaline, 10 mg/mL + 5 μg/mL). A 1–2 cm incision was made through the skin and fascia of the targeted muscle. Using a 6 mm Pelomi needle (Bergström technique) with manual suction, approximately 200 mg of muscle tissue was collected. Following extraction, the biopsies were rinsed in ice‐cold saline solution (0.9% NaCl) and carefully trimmed to remove visible fat, connective tissue, and blood before being snap frozen in isopentane pre‐cooled in dry ice. All samples were stored at −80°C until further analyses.

### 2.10 Immunohistochemistry

Immunohistochemistry was performed as previously described (Vikmoen et al. 2025). Briefly, cross‐sections (8 μm; cut at −20°C, CM3050; Leica Microsystems GmbH, Wetzlar, Germany) were mounted on microscope slides (Superfrost Plus; Thermo Fisher Scientific Inc., Waltham, MA, USA), air‐dried, and stored at −80°C. After thawing, sections were blocked for 60 min in 1% BSA (cat# A4503; Sigma‐Aldrich Corp., St Louis, MO, USA), 1% fat‐free dry milk and 0.05% PBS‐T solution (cat#524650; Calbiochem, EMD Biosciences Inc., San Diego, CA, USA). Slides were incubated overnight at 4°C with antibodies against CD31 (1:100; clone JC70A; M0823; Dako A/S, Glostrup, Denmark) together with MyHCI (BA‐F8; 1:500; DSHB Hybridoma Product BA‐F8) and dystrophin (1:500; cat# ab15277) in blocking solution. Following PBS‐T (0.05%) washes (3 × 5 min), sections were incubated with appropriate secondary antibodies (Alexa Fluor‐conjugated secondary antibodies, Invitrogen, CA) in blocking solution for 1 h at room temperature and mounted with ProLong Gold Antifade (cat# P10144, Invitrogen Molecular Probes, Eugene, OR, USA). Images were acquired using an Olympus BX61 microscope with a DP72 camera. Capillary indices were quantified using TEMA software (CheckVision, Denmark) and expressed as capillaries around each fiber (CAF) and capillaries per fiber area (CAFA), separately for type I and type II fibers.

### 2.11 Immunoblotting

Approximately 50 mg of muscle tissue was homogenized and fractionated into cytosolic, nuclear, membrane, and cytoskeletal fractions using the ProteoExtract Subcellular Proteome Extraction Kit (Calbiochem, EMD Millipore Corporation, Billerica, MA, USA), according to the manufacturer's instructions. Protein concentration in each fraction was determined using the DC Protein Assay (Reagent A, B and S; 5000113, 5000114, 5000115; Bio‐Rad, CA, USA) and Multiskan FC Microplate Photometer (version 1.01.16; Thermo Scientific, MA, USA), with samples measured in triplicate against a standard curve. Immunoblot analyses were performed on the cytosolic and membrane fractions. Proteins extracted from muscle samples were analyzed by immunoblotting. All samples were diluted with 4×Laemmli Sample Buffer (16100747; Bio‐Rad, CA, USA), 5M DTT (1610610; Bio‐Rad, CA, USA) and Type 1 water. Equal amounts of protein were loaded per well (6–24 μg depending on subcellular fraction) and separated under denatured conditions on 4%–20% Mini‐PROTEAN TGX Stain‐Free Protein Gels, 10 well, 50 μL (4568094; Bio‐Rad, CA, USA) for 30–40 min at 200 V using 10× Tris/Glycine/SDS Running buffer (1610772; Bio‐Rad, CA, USA). Proteins were transferred to Immun‐Blot PVDF membranes (1620177; Bio‐Rad, CA, USA) using a *Trans*‐Blot Turbo Transfer System (1704150; Bio‐Rad, CA, USA) with the mixed MW program (25 V for 7 min). Membranes were blocked in 5% fat‐free skimmed milk (1.15363; Millipore, Merck KGaA, Darmstadt, Germany) in 0.1% TBS‐T (10× Tris Buffered Saline 1706435; Bio‐Rad, CA, USA; Tween20 P9416; Sigma‐Aldrich, Merck KGaA, Darmstadt, Germany) at room temperature for 2 h. After blocking, membranes were divided into pieces based on molecular weight (Precision Plus Protein All Blue Prestained Protein Standards; 1610373; Bio‐Rad, CA, USA) and incubated overnight at 4°C with primary antibodies targeting ATG5 (1:1000, cat # 12994, Cell Signaling Technologies, Danvers, MA, USA), p62 (1:2000, cat # 56416, Abcam, Cambridge, UK), BCL2 (1:1000, cat # 4223, Cell Signaling Technologies, Danvers, MA, USA), LC3B1 (1:1000, cat # 2775, Cell Signaling Technologies, Danvers, MA, USA), HSP60 (1:4000, cat # ADI‐SPA‐807, Enzo Life Sciences Inc., Farmingdale, NY, US), and HSP70 (1:4000, cat # ADI‐SPA‐810, Enzo Life Sciences Inc., Farmingdale, NY, US). The next day, membranes were washed in 0.1% TBS‐T for 15 min and then 3 × 5 min in TBS before incubation with HRP‐linked secondary antibody for 1 h at room temperature (Goat anti‐Rabbit IgG, 1:30000, 31460; Invitrogen, IL, USA). All antibodies were diluted in 1% fat‐free skimmed milk in 0.1% TBS‐T. After secondary incubation, membranes were washed in TBS‐T and TBS solutions. Bands were visualized using an HRP detection system (Thermo Scientific SuperSignal West Pico PLUS Chemiluminescent Substrate, 15659364; Thermo Scientific, MA, USA) and imaged using a ChemiDoc MP Image System. Chemiluminescence was quantified using Image Lab Software (ver. 6.1.0; Bio‐Rad Laboratories Inc., CA, USA). Band densities were normalized to total lane protein quantified from stain‐free gel imaging prior to statistical analysis.

### 2.12 Disease and Treatment

Details on oncological treatment (type and dose), disease stage, and comorbidity were obtained from medical records and have been reported previously (Vikmoen et al. 2024). Relative dose intensity (RDI) was calculated according to Longo et al. (1991). In addition, the percentage of planned dose administered was calculated for docetaxel, epirubicin, and cyclophosphamide, and for all treatments combined (Vikmoen et al. 2024).

### 2.13 Statistical Analysis

**FIGURE 2:**
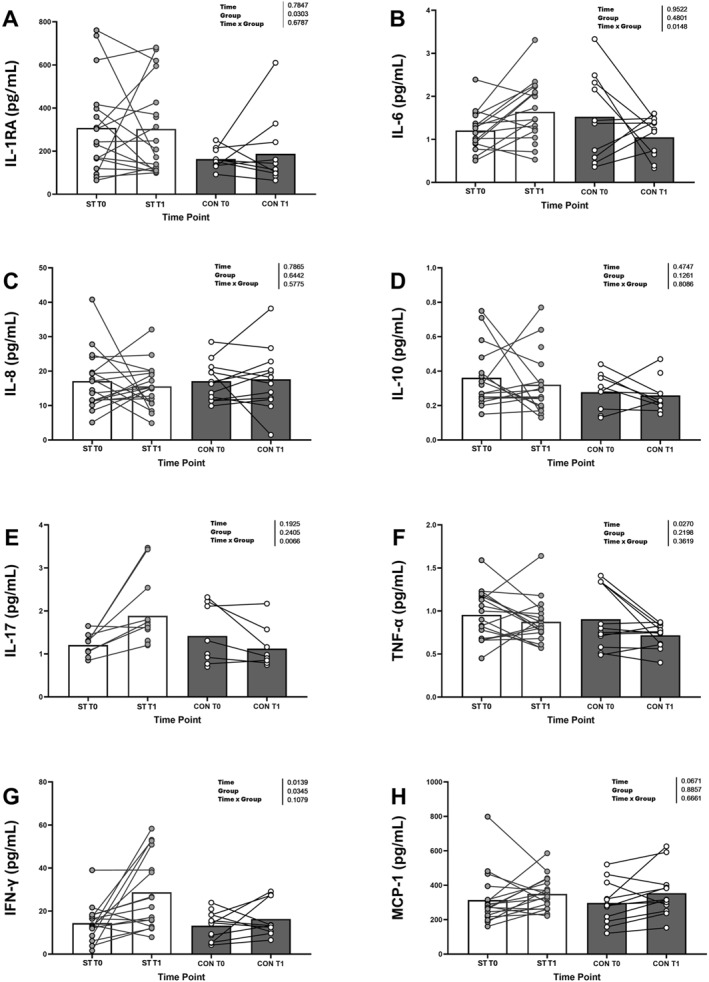
Serum cytokine concentrations pre‐ and post‐chemotherapy. Individual values and mean ± SD for serum cytokines at baseline (T0) and post‐intervention (T1) in the strength training (ST; white) and usual care control (CON; gray) groups: (A) IL‐1RA (ST *n* = 16, CON *n* = 9), (B) IL‐6 (ST *n* = 16, CON *n* = 10), (C) IL‐8 (ST *n* = 18, CON *n* = 12), (D) IL‐10 (ST *n* = 15, CON *n* = 9), (E) IL‐17 (ST *n* = 9, CON *n* = 8), (F) TNF‐α (ST *n* = 18, CON *n* = 12), (G) IFN‐γ (ST *n* = 17, CON *n* = 12), and (H) MCP‐1 (ST *n* = 18, CON *n* = 12), n indicates the number of participants with paired T0 and T1 values plotted for each cytokine. *p*‐values shown in each panel are from two‐way mixed‐effects models (REML) testing fixed effects of time, group, and the group×time interaction; the group×time interaction corresponds to the between‐group difference in change from T0 to T1 (ΔΔ). Estimates of ΔΔ with 95% confidence intervals are reported in the results.

**FIGURE 3:**
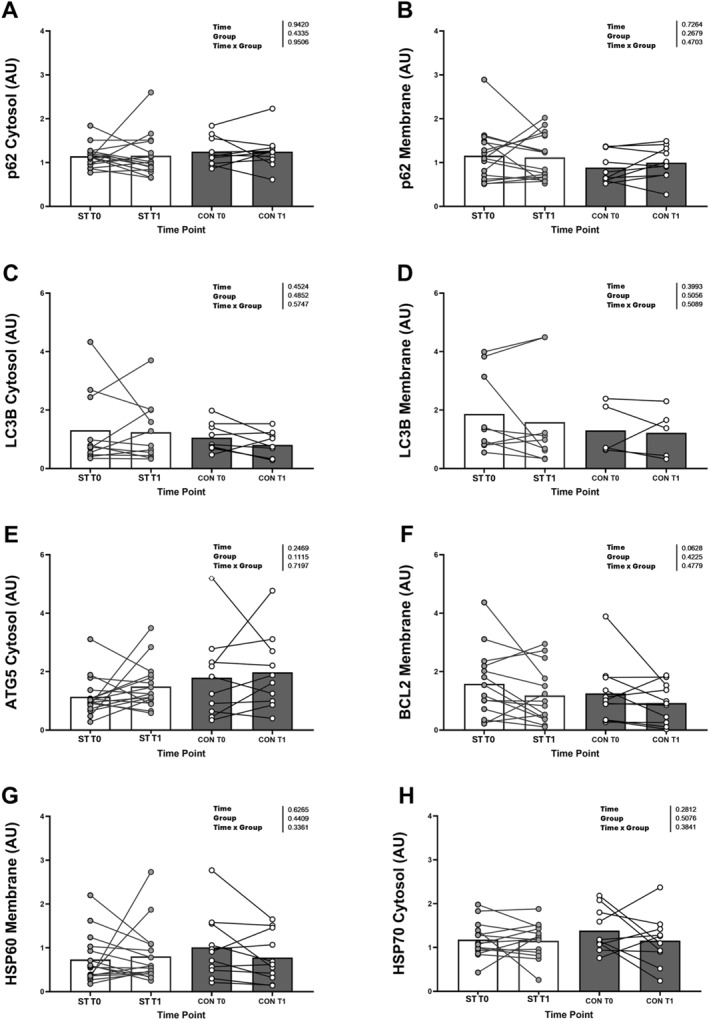
Skeletal muscle autophagy‐ and heat‐shock‐related proteins pre‐ and post‐chemotherapy. Individual values and mean ± SD for protein abundance (arbitrary units, AU) at baseline (T0) and post‐intervention (T1) in the strength training (ST; white) and usual care control (CON; gray) groups: (A) p62 cytosol (ST *n* = 14, CON *n* = 10), (B) p62 membrane (ST *n* = 16, CON *n* = 10), (C) LC3B1 cytosol (ST *n* = 12, CON *n* = 9), (D) LC3B1 membrane (ST *n* = 12, CON *n* = 5), (E) ATG5 cytosol (ST *n* = 16, CON *n* = 10), (F) BCL2 membrane (ST *n* = 15, CON *n* = 10), (G) HSP60 membrane (ST *n* = 16, CON *n* = 10), and (H) HSP70 cytosol (ST *n* = 14, CON *n* = 10). Protein abundance was normalized to total lane protein. *n* indicates the number of participants with paired T0 and T1 values plotted for each outcome. *p*‐values shown in each panel are from two‐way mixed‐effects models (REML) testing fixed effects of time, group, and the group×time interaction; the group×time interaction corresponds to the between‐group difference in change from T0 to T1 (ΔΔ).

Data are presented as mean ± SD unless otherwise stated. Normality of model residuals was assessed using the Shapiro‐Wilk test. To evaluate differences between groups (strength training vs. control) over time (T0 vs. T1), outcomes were analyzed using a two‐way mixed‐effects model (REML) with fixed effects for group, time, and the group × time interaction, implemented in GraphPad Prism (version 10.3.1). Analyses were conducted using a modified intention‐to‐treat (mITT) approach: participants were analyzed in their randomized groups, and mixed‐effects models incorporated all available outcome observations without imputation. This likelihood‐based approach accommodates unbalanced repeated‐measures data under a missing‐at‐random assumption. Mixed‐effects models used all available observations and did not require measurements at both time points; however, for visualization (Figures 2, 3), only participants with values at both T0 and T1 for the relevant analyte are shown. The primary test of the intervention effect was the group×time interaction. Intervention effects are reported as the between‐group difference in change from T0 to T1 (ΔΔ; interaction estimate) with 95% confidence intervals; positive ΔΔ values indicate a greater increase (or smaller decrease) over time in the strength‐training group compared with control. No post‐hoc multiple‐comparisons tests were performed; within‐group patterns of change are described descriptively. Given the number of secondary endpoints assessed, *p*‐values should be interpreted cautiously. For serum cytokines, values below the assay lower limit of quantification (LLOQ) were considered not quantifiable and were treated as missing for that analyte, consequently, sample size varied across cytokines. Similarly, sample size varied across muscle protein analyses because biopsy material and technical availability differed across targets. Exploratory correlation analyses (Pearson's r) assessed relationships between changes (T1‐T0) in physiological outcomes and biomarker responses, and interrelationships among cytokine changes, using pooled data across both groups. Because pooling may induce associations driven by between‐group differences, correlation findings were considered hypothesis‐generating and correlation *p*‐values were interpreted descriptively (no formal adjustment for multiple comparisons). Statistical significance was set at *p* < 0.05 for model‐based tests.

## 3 Results

Anthropometrics, co‐morbidities, tumor stage, HER2 status, treatment variables as well as physical performance outcomes, have been reported previously (Vikmoen et al. 2024) and the groups were comparable at baseline. In a subsequent study from the same cohort (Vikmoen et al. 2025), VO_2peak_ declined in both groups as did hemoglobin, however, aerobic enzyme content was preserved with strength training and declined in the CON group. In the CON group, type II fiber capillary density declined, while physical fatigue increased (Vikmoen et al. 2025). Sample sizes differed by analyte because some cytokine values were below the assay lower limit of quantification (LLOQ) and because sample availability was limited for some muscle protein analyses.

### 3.1 Cytokine Response

The mixed‐effects model showed that changes over time differed between groups for IL‐6 and IL‐17. For IL‐6, the between‐group difference in change (ΔΔ) was 0.90 pg/mL (95% CI 0.19 to 1.61; group×time interaction *p* = 0.0148), reflecting an increase in the strength‐training group (T0 1.21 ± 0.47 to T1 1.69 ± 0.71 pg/mL) and a decrease in the control group (T0 1.53 ± 1.01 to T1 1.15 ± 0.43 pg/mL) (Figure 2B). For IL‐17, ΔΔ was 1.08 pg/mL (95% CI 0.36 to 1.80; *p* = 0.0066), with IL‐17 increasing in the strength‐training group (T0 1.24 ± 0.25 to T1 2.18 ± 0.87 pg/mL) and decreasing in the control group (T0 1.61 ± 0.69 to T1 1.25 ± 0.53 pg/mL) (Figure 2E). For IFN‐γ, there was no statistically significant group×time interaction (ΔΔ 10.56 pg/mL, 95% CI ‐2.49 to 23.60; *p* = 0.1079); descriptively, IFN‐γ increased in the strength‐training group (T0 14.4 ± 8.6 to T1 28.8 ± 17.3 pg/mL), whereas the control group showed little change (Figure 2G). No evidence of change over time was observed for IL‐1RA, IL‐8, IL‐10, TNF‐α, or MCP‐1 (all group×time interaction *p* > 0.05) (Figure 2A,C,D,F, and H). Baseline, post‐intervention, and change values for all cytokines together with analyte‐specific sample sizes, are provided in Table S1.

### 3.2 Autophagy and Heat‐Shock Related Proteins

No evidence of change over time (group×time interaction *p* > 0.05) was observed for any of the measured proteins (ATG5, p62, BCL2, LC3B1, HSP60, or HSP70) (Figure 3).

### 3.3 Exploratory Correlation Analyses

**FIGURE 4:**
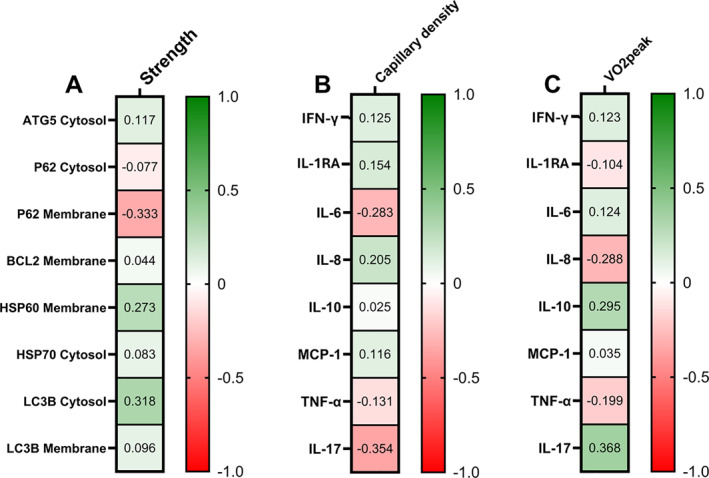
Exploratory correlations between changes in physiological outcomes and biomarker responses. Heatmaps show Pearson correlation coefficients (r) between changes from T0 to T1 (T1‐T0) in (A) muscle strength and selected muscle proteins, (B) type II fiber capillary density and cytokines, and (C) VO_2peak_ and cytokines. Shading reflects the direction and magnitude of correlations (scale −1 to 1). Values represent correlation coefficients. Correlations were computed using pooled data across both groups and are presented for hypothesis generation; pooling may introduce associations driven by between‐group differences. N varies across analyte pairs (range 11–28) due to missing values.

**FIGURE 5:**
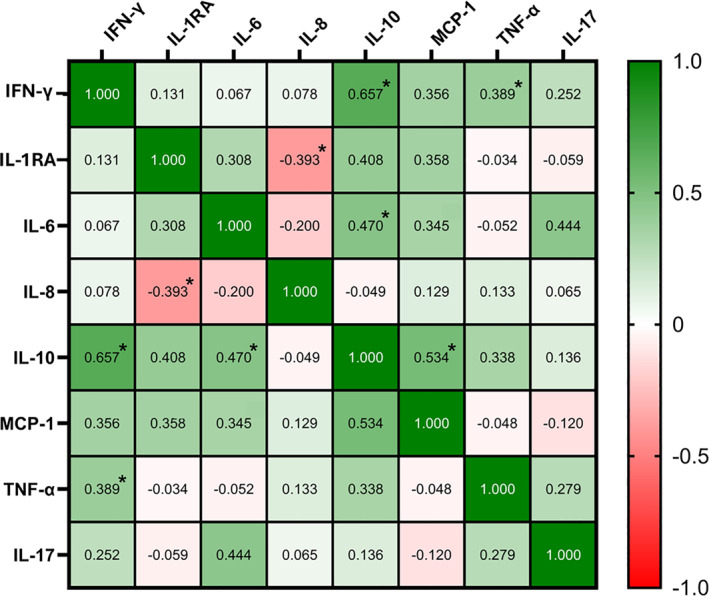
Exploratory interrelationships among cytokine changes. Heatmap shows Pearson correlation coefficients (r) between changes from T0 to T1 (T1‐T0) in serum cytokines. Shading reflects the direction and magnitude of correlations (scale −1 to 1). Values represent correlation coefficients; * indicates nominal *p* < 0.05 (uncorrected). Analyses are hypothesis‐generating and *p*‐values are interpreted descriptively given multiple testing. N varies across cytokines (range 13–28) due to values below the LLOQ and other missing data.

We did not observe any associations between changes in muscle strength and changes in any of the autophagy/heat‐shock‐related proteins (Figure 4A). Similarly, no associations were observed between changes in capillary density and changes in cytokine concentrations (Figure 4B) or between changes in VO_2peak_ and changes in cytokine concentrations (Figure 4C). We also examined exploratory interrelationships among changes in cytokines (Figure 5). Several cytokine pairs showed nominal (uncorrected) correlations at *p* < 0.05, including positive correlations between IFN‐γ and IL‐10 and between IFN‐γ and TNF‐α, as well as between IL‐6 and IL‐10 and between MCP‐1 and IL‐10. A nominal negative correlation was observed between IL‐1RA and IL‐8. In addition, given multiple testing, these correlation *p*‐values are interpreted descriptively.

## 4 Discussion

The present study investigated how supervised strength training during chemotherapy influences serum cytokine concentrations and skeletal muscle autophagy‐ and heat‐shock‐related proteins in women with breast cancer. Using mixed‐effects models, we observed evidence of change over time between groups for IL‐6 and IL‐17 (group×time interactions), with IL‐6 and IL‐17 increasing in the strength training group and decreasing in the control group. For IFN‐γ, the group×time interaction was not statistically significant, descriptively, IFN‐γ increased in the strength training group while the control group showed little to no change. No evidence of change over time was observed for the remaining cytokines or for any measured autophagy‐ or heat‐shock‐related proteins. Exploratory correlation analyses did not show any associations between changes in muscle strength and muscle proteins, changes in VO_2peak_ and cytokines, or changes in capillary density and cytokines. Nominal positive correlations were observed between changes in IFN‐γ and IL‐10 and between changes in IFN‐γ and TNF‐α, as well as between IL‐6 and IL‐10 and between MCP‐1 and IL‐10. A nominal negative correlation was observed between IL‐1RA and IL‐8.

In the present study, we selected a serum cytokine panel to capture key aspects of systemic immune signaling. IL‐6 and TNF‐α were selected as cytokines commonly linked to inflammatory signaling and cancer‐related symptoms, including muscle wasting and fatigue, whereas IL‐10 and IL‐1RA were included as anti‐inflammatory regulators (Cole et al. 2018; Webster et al. 2020). IL‐17 and IFN‐γ, primarily secreted by Th17 and Th1 cells respectively, were included due to their roles in coordinating tissue repair, immune activation, and possible antitumor effects (McGeachy et al. 2019; Zaidi 2019). MCP‐1 and IL‐8 were selected for their involvement in monocyte recruitment and angiogenesis, processes that may also be influenced by exercise (Cerqueira et al. 2019; Cole et al. 2018). Systemic inflammation has been reported to increase during cancer treatment (Cengiz et al. 2001; Lyon et al. 2016; Schauer et al. 2021; Wang et al. 2012). In contrast, in the present study IL‐6 and IL‐17 decreased in the CON group but increased in the strength training group, consistent with group×time interactions indicating divergent cytokine trajectories between groups. These findings should be interpreted cautiously. Although IL‐6, IL‐17, and IFN‐γ are often described as pro‐inflammatory cytokines, circulating levels of these markers do not by themselves show whether the response is harmful, adaptive, or clinically meaningful. In the context of repeated resistance exercise performed during chemotherapy, the observed pattern may reflect exercise‐related immune signaling occurring alongside ongoing treatment‐related stress, rather than clear evidence of either worsening systemic inflammation or improved antitumor immunity.

Among the cytokines examined, IL‐6 is of particular interest because it can have different roles depending on the physiological context. IL‐6 released from contracting muscle acts as a myokine (Muñoz‐Cánoves et al. 2013), and acute exercise‐induced IL‐6 can promote an anti‐inflammatory cascade that upregulates IL‐10 and IL‐1RA and suppresses TNF‐α signaling (Pedersen and Febbraio 2012; Starkie et al. 2003). Consistent with this idea, we observed a nominal (uncorrected) positive correlation between changes in IL‐6 and IL‐10, which may align with exercise‐related anti‐inflammatory signaling pathways (Pedersen and Febbraio 2012; Starkie et al. 2003) although no corresponding correlation was observed between IL‐6 and IL‐1RA. Importantly, because blood samples were collected at least 48 h after the final scheduled exercise session, the present data do not capture acute post‐exercise cytokine kinetics. Instead, the higher IL‐6 concentration observed at T1 in the strength training group may reflect more sustained shifts in circulating cytokines during active treatment. However, without repeated sampling or complementary immune phenotyping, the physiological meaning of this finding remains uncertain. In breast‐cancer survivors who had completed therapy, exercise has been associated with reduced IL‐6 concentrations (Bettariga et al. 2025), suggesting that treatment status and the inflammatory milieu during chemotherapy may influence the direction of cytokine responses.

IL‐17 may also reflect different biological processes depending on the context. Although it is commonly viewed as a pro‐inflammatory cytokine (Zenobia and Hajishengallis 2015), it also contributes to barrier defense and coordinated tissue repair following injury (McGeachy et al. 2019). In the present study, IL‐17 increased in the strength‐training group while it declined in usual care, consistent with a significant group×time interaction. Because some IL‐17 values were below the assay LLOQ, IL‐17 results are based on fewer quantifiable observations than several other cytokines and should be interpreted accordingly. Evidence on exercise effects on IL‐17 is limited. In one study, endurance exercise performed in a hot environment increased IL‐17, whereas the same exercise in neutral conditions did not (Satarifard et al. 2012), suggesting that additional physiological stressors may modulate Th17‐related responses. During chemotherapy, oxidative stress and DNA‐damage signaling are elevated (Abimannan et al. 2016), which could plausibly influence immune responsiveness. One possible interpretation is that the observed IL‐17 increase reflects a combined effect of repeated contractile stimuli and the physiological stress of ongoing chemotherapy, rather than necessarily indicating a harmful increase in inflammation; however, this interpretation should be considered preliminary because immune cell phenotypes and local inflammatory signaling were not assessed. Notably, IL‐17 has been implicated in repair‐associated processes including angiogenesis in certain contexts (McGeachy et al. 2019), which may be compatible with the preserved skeletal muscle capillary density observed with training in this cohort. However, we did not observe any associations between changes in cytokines and capillary density.

In the present study, IFN‐γ showed a descriptive increase in the strength training group, whereas the control group changed little; however, there was no statistically significant group×time interaction, and this pattern should be interpreted cautiously. Although IFN‐γ is often classified as a pro‐inflammatory Th1 cytokine, it can also trigger feedback mechanisms that limit inflammation, including induction of IL‐10 under certain conditions (Flaishon et al. 2002). Evidence regarding exercise‐related changes in IFN‐γ is mixed. IFN‐γ has been reported to decrease in response to moderate physical activity during neoadjuvant chemotherapy in breast cancer patients (Garrone et al. 2024) and to remain unchanged following 24 weeks of concurrent training in healthy young individuals (Vázquez‐Lorente et al. 2025). IFN‐γ is also recognized for its role in antitumor immunity (Zaidi 2019) however, because tumor‐related outcomes and immune cell phenotypes were not assessed in the present study, the clinical significance of increased circulating IFN‐γ levels cannot be determined. Accordingly, the observed pattern likely reflects treatment‐related immune signaling during strength training rather than a specific antitumor response. In contrast to some studies in non‐cancer populations where exercise interventions have been associated with changes in other circulating cytokines such as TNF‐α or IL‐10 (Cerqueira et al. 2019), we observed no evidence of change over time for these markers, and no changes for other cytokines beyond IL‐6 and IL‐17. This may reflect the complexity of immune regulation during chemotherapy and/or methodological factors, including limited power for some markers and the possibility that cytokine responses occurred outside the post‐intervention sampling window. From a clinical perspective, these findings should be interpreted cautiously. They do not suggest that strength training worsened inflammation or promoted tumor growth, but neither do they support a direct enhancement of antitumor immunity. Instead, they point to altered circulating immune signaling during treatment, the clinical relevance of which remains unclear.

We found no evidence of change over time (group × time interaction *p* > 0.05) in the autophagy‐ and heat‐shock‐related proteins ATG5, p62, BCL2, LC3B1, HSP60, or HSP70. This suggests that the beneficial effects of strength training on muscle‐related outcomes observed previously in this cohort were not accompanied by detectable changes in the selected intramuscular stress‐response markers at the time points assessed in the present study. Autophagy is a key intracellular degradation pathway that contributes to muscle homeostasis during cellular stress (Gómez‐Virgilio et al. 2022; Ryter et al. 2014) and dysregulation of autophagy has been proposed to contribute to muscle wasting through accumulation of damaged proteins and organelles (Sandri 2013). However, interpretation of autophagy in human muscle based on static protein abundance is challenging, as autophagic flux can change quickly and may not be captured by single time‐point measurements. Therefore, the lack of change in these markers does not rule out changes in autophagy function during the intervention. These null findings may seem inconsistent with the improvements in muscle strength and preservation of CSA observed in the ST group in this cohort (Vikmoen et al. 2025, 2024). Several factors may explain this. First, the biopsy timing may not have captured the period when autophagy‐ or heat‐shock‐related signaling changes are most evident. Second, exercise‐related protection against declines in muscle function during chemotherapy may be mediated through other mechanisms not measured in this study. Third, effects on these specific proteins may have been modest under chemotherapy‐related stress and/or limited by statistical power for some targets. Future studies incorporating additional sampling time points, including time points closer to the final training session, may better capture changes in cytokines and intramuscular signaling. Moreover, exploratory correlation analyses did not show any linear relationships between changes in muscle strength and autophagy‐related proteins, or between changes in VO_2peak_ or capillary density and changes in the measured cytokines. Together, these findings suggest that, within the limits of the present dataset and sampling schedule, improvements in physical performance and skeletal muscle structure observed with training in this cohort (Vikmoen et al. 2024), were not accompanied by corresponding relationships with the selected circulating cytokines or autophagy‐related markers.

In the present study, we focused specifically on strength training rather than a mixed exercise mode. Much of the existing oncology exercise literature combines aerobic and resistance training (de Hoop et al. 2024), which makes mechanistic interpretation difficult. By isolating strength training, the present study provides more mode‐specific information. This is relevant because resistance training differs from aerobic exercise in contraction pattern, metabolic demand, and the signaling pathways likely to be engaged, and it specifically targets muscle mass and strength, which are highly relevant outcomes during chemotherapy (Freedman et al. 2004; Saquib et al. 2007; Van Den Berg et al. 2017; Vance et al. 2011). At the same time, this design also limits direct comparison with mixed‐modality interventions. Future studies directly comparing resistance, aerobic, and combined training during chemotherapy would help clarify whether circulating cytokine responses and intramuscular stress‐related signaling differ by exercise modality.

## 5 Strength and Limitations

This study has several strengths, including the randomized controlled design, supervised high‐load strength training delivered during chemotherapy, and the integration of both systemic and muscle‐specific outcomes. In addition, complementary data from the same cohort (Vikmoen et al. 2025, 2024) demonstrated preservation of aerobic enzyme content and capillary density with strength training, supporting beneficial effects on muscle oxidative capacity and capillary supply. Some limitations should be acknowledged. For cytokines, some values were below the assay lower limit of quantification (LLOQ), and fewer muscle biopsies were available for certain autophagy‐related proteins; consequently, analyte‐specific sample sizes varied and statistical power to detect modest effects may have been reduced. Because values below the LLOQ may not occur at random, this could also introduce uncertainty for specific cytokines with lower quantifiable coverage. Our focus on a selected panel of cytokines and autophagy/heat‐shock markers, while biologically relevant, does not capture the full complexity of molecular adaptations in muscle, additional targets (e.g., markers of mitochondrial biogenesis, mitophagy, or anabolic/catabolic signaling pathways) may yield further insight. Moreover, the single post‐intervention assessment may have missed temporary fluctuations in cytokine levels or autophagy‐related proteins. We did not perform a per‐protocol analysis in the present study. Although this was done in a previous paper from the same cohort, the analyte‐specific sample sizes for the cytokine and muscle protein outcomes reported here were more limited. We therefore retained the modified intention‐to‐treat approach to preserve the available data, as a per‐protocol analysis would likely have further reduced interpretability. Although adherence to the training program was good, variability in chemotherapy regimens, side effects, and individual recovery capacity may have influenced exercise tolerance and biomarker responses. Finally, we were not able to determine whether biomarker changes were associated with pathological response to neoadjuvant chemotherapy, such as residual disease burden or pathological complete response at surgery. Future studies should examine this more directly.

## 6 Conclusions

In this randomized controlled trial, supervised strength training during chemotherapy in women with breast cancer was associated with increased serum IL‐6 and IL‐17 compared with usual care, without changes in skeletal muscle autophagy‐ or heat‐shock related proteins at the assessed time points. These findings indicate that strength training during active treatment can coincide with measurable shifts in circulating cytokines, whereas selected intramuscular markers of autophagy and cellular stress remained unchanged. The clinical and mechanistic significance of these cytokine shifts remains uncertain, particularly given the complexity of immune regulation during chemotherapy and the limited sampling time points. Future studies should incorporate additional sampling time points, broader immune and muscle signaling panels (including mitochondrial and anabolic/catabolic pathways), and clinically relevant endpoints to clarify whether these cytokine responses relate to muscle function, treatment tolerance, recovery, pathological response or survivorship outcomes. Larger randomized trials with longer follow‐up are warranted to validate and extend these findings. In addition, future studies with larger samples may benefit from complementary per‐protocol analyses.

## Author Contributions

T.R. and S.B. contributed to the concept and design of the study. E.S., K.V.S., O.V., A.H., H.N.Ø. and A.W.S., and performed data collection and analyses. O.V. and E.S. performed statistical analyses. Project administration was carried out by E.S., K.V.S. and A.H. E.S. drafted the original manuscript, and all authors (E.S., K.V.S., O.V., A.H., H.N.Ø., A.W.S., S.B., T.R.) contributed to manuscript review and editing. T.R. was responsible for funding acquisition.

## Conflicts of Interest

The authors declare no conflicts of interest.

## Supporting information


**Table S1:** Serum cytokine outcomes.

## Data Availability

The data that support the findings of this study are available on request from the corresponding author.
