# Supplementary material for: The Effect of Strength Training During Chemotherapy in Women With Breast Cancer on Serum Cytokine Concentrations and Skeletal Muscle Autophagy‐Related Proteins
Source: Eur J Sport Sci. 2026 Jun 25;26(7):e70213. doi: 10.1002/ejsc.70213 (PMC13303106; doi:10.1002/ejsc.70213)
Supplement: Supplementary file 1 — Table S1: Serum cytokine outcomes. [file EJSC-26-e70213-s001.docx]

Supplementary table 1. Serum cytokine outcomes

| Outcome | Group | n paired | T0 mean ± SD | T1 mean ± SD | Change (T1-T0)  mean ± SD | ΔΔ (95% CI) | Group^x^time p |
| --- | --- | --- | --- | --- | --- | --- | --- |
| IFN-γ (pg/mL) | ST | 16 | 14.41 ± 8.57 | 28.79 ± 17.33 | 14.38 ± 17.33 | 10.56 (-2.49 to 23.60) | 0.1079 |
|  | CON | 10 | 12.83 ± 6.66 | 16.32 ± 8.35 | 3.48 ± 10.06 |  |  |
|  |  |  |  |  |  |  |  |
| IL-1RA (pg/mL) | ST | 17 | 307.9 ± 225.5 | 299.7 ± 219.1 | -8.18 ± 240.1 | -35.53 (-210.5 to 139.4) | 0.6787 |
|  | CON | 9 | 166.4 ± 49.1 | 206.8 ± 172.1 | 40.33 ± 165.2 |  |  |
|  |  |  |  |  |  |  |  |
| IL-6 (pg/mL) | ST | 16 | 1.21 ± 0.47 | 1.69 ± 0.71 | 0.48 ± 0.64 | 0.90 (0.19 to 1.61) | 0.0148 |
|  | CON | 10 | 1.53 ± 1.01 | 1.15 ± 0.43 | -0.38 ± 1.17 |  |  |
|  |  |  |  |  |  |  |  |
| IL-8 (pg/mL) | ST | 18 | 17.12 ± 8.72 | 15.58 ± 6.52 | -1.54 ± 11.03 | -2.07 (-9.60 to 5.46) | 0.5775 |
|  | CON | 12 | 17.09 ± 6.95 | 17.63 ± 9.27 | 0.53 ± 7.74 |  |  |
|  |  |  |  |  |  |  |  |
| IL-10 (pg/mL) | ST | 16 | 0.36 ± 0.14 | 0.31 ± 0.17 | -0.05 ± 0.24 | -0.02 (-0.20 to 0.16) | 0.8086 |
|  | CON | 8 | 0.30 ± 0.14 | 0.26 ± 0.09 | -0.04 ± 0.13 |  |  |
|  |  |  |  |  |  |  |  |
| MCP-1 (pg/mL) | ST | 18 | 314.1 ± 127.9 | 349.6 ± 95.2 | 35.5 ± 143.6 | -21.08 (-120.1 to 77.94) | 0.6661 |
|  | CON | 12 | 297.7 ± 121.7 | 354.2 ± 138.1 | 56.6 ± 104.8 |  |  |
|  |  |  |  |  |  |  |  |
| TNF-α (pg/mL) | ST | 18 | 0.96 ± 0.37 | 0.87 ± 0.25 | -0.08 ± 0.31 | 0.11 (-0.13 to 0.34) | 0.3619 |
|  | CON | 12 | 0.90 ± 0.24 | 0.72 ± 0.14 | -0.19 ± 0.30 |  |  |
|  |  |  |  |  |  |  |  |
| IL-17 (pg/mL) | ST | 9 | 1.24 ± 0.25 | 2.18 ± 0.87 | 0.94 ± 0.84 | 1.08 (0.36 to 1.80) | 0.0066 |
|  | CON | 8 | 1.61 ± 0.69 | 1.25 ± 0.53 | -0.36 ± 0.48 |  |  |

Values are mean ± SD. Change was calculated as T1 - T0. ΔΔ indicates the between-group difference in change from T0 to T1 (strength training minus control) from the mixed-effects model, with 95% confidence interval. Values below the assay lower limit of quantification were treated as missing for that analyte.
